# Gaps and recommendations for clinical management of truncal acne from the Personalising Acne: Consensus of Experts panel

**DOI:** 10.1016/j.jdin.2021.06.007

**Published:** 2021-08-17

**Authors:** Jerry Tan, Andrew Alexis, Hilary Baldwin, Stefan Beissert, Vincenzo Bettoli, James Del Rosso, Brigitte Dréno, Linda Stein Gold, Julie Harper, Charles Lynde, Diane Thiboutot, Jonathan Weiss, Alison M. Layton

**Affiliations:** a Windsor Clinical Research Inc, Windsor, Ontario, Canada; b Department of Medicine, University of Western Ontario, Windsor Campus, Windsor, Ontario, Canada; c Weill Cornell Medicine, New York, New York; d Robert Wood Johnson Medical Center, New Brunswick, New Jersey; e The Acne Treatment and Research Center, Brooklyn, New York; f Department of Dermatology, University Hospital Carl Gustav Carus, TU Dresden, Dresden, Germany; g Dermatology Unit – Teaching Hospital, Azienda Ospedaliera, University of Ferrara, Ferrara, Italy; h Thomas Dermatology, Las Vegas, Nevada; i JDR Dermatology Research, Las Vegas, Nevada; j Dermato-cancérology Department, CHU Nantes, University of Nantes, Nantes, France; k Henry Ford Health System, Detroit, Michigan; l Dermatology and Skin Care Center of Birmingham, Birmingham, Alabama; m Department of Medicine, University of Toronto, Markham, Ontario, Canada; n Lynderm Research Inc, Markham, Ontario, Canada; o Department of Dermatology, Pennsylvania State University College of Medicine, Hershey, Philadelphia; p Georgia Dermatology Partners, Snellville, Georgia; q Hull York Medical School, University of York, York, United Kingdom; r Harrogate and District NHS Foundation Trust, United Kingdom

**Keywords:** acne vulgaris, back acne, chest acne, consensus, Delphi process, shoulder acne, truncal acne, PACE, Personalising Acne: Consensus of Experts, PGA, physicians' global assessment

## Abstract

**Background:**

Truncal acne is common and burdensome for patients; however, there is paucity of evidence and guidance for the management of truncal acne. Currently, clinical practice guidelines provide very little guidance on the assessment or management of truncal acne.

**Objectives:**

To identify unmet needs in truncal acne and make recommendations to address clinical and management gaps using an international consensus.

**Methods:**

The Personalising Acne: Consensus of Experts panel consisted of 13 dermatologists, who used a modified Delphi approach to reach a consensus on statements related to clinically relevant aspects of truncal acne evaluation and management. A consensus was defined as ≥75% of the panelists voting “agree” or “strongly agree.” The voting was electronic and blinded.

**Results:**

The panel identified gaps and made recommendations related to truncal acne identification, assessment, and grading; the evaluation of the impact on patients; and treatment goals and factors to be considered for its management.

**Limitations:**

The recommendations are based on expert opinion, in the absence of high-quality evidence.

**Conclusions:**

We highlighted addressing not just facial acne but also truncal acne during patient consultations. The recommendations made herein may help facilitate the care of patients who present with truncal acne, with or without facial acne.

**Capsule Summary**

• Truncal acne is common and burdensome for patients; however, published evidence and guidance for its clinical management are lacking.
• We provide recommendations for relevant clinical factors to be considered when managing patients with truncal acne, to improve comprehensive patient care.

## Introduction

Acne vulgaris is estimated to affect 9.4% of the global population (for all ages). Nearly three-quarters (73%) of adults (over 20 years of age) report ever having acne,^1^, ^2^, ^3^ with as many as 61% presenting with truncal involvement.^4^, ^5^, ^6^, ^7^, ^8^ The latter is inclusive of the shoulders, chest, and back.^9^ Despite the high prevalence of truncal acne, there is paucity of data on its clinical aspects, including management options. The treatment of truncal acne has not been rigorously studied in clinical trials, with only 1 publication reporting the results of 2 identical vehicle-controlled randomized clinical trials in patients with moderate facial and truncal acne.^10^ Furthermore, limited information is provided by clinical guidelines about its assessment and grading, factors to be considered during discussions with patients, and its treatment.^11^, ^12^, ^13^ This lack of evidence and guidance, coupled with the fact that many patients do not voluntarily report their truncal acne, is likely to result in undertreatment and unresolved disease-related burdens.^4^

With acne well-established as a condition with adverse psychosocial impact,^14^^,^^15^ there is a need to address and manage the whole spectrum of acne presentations, including that on the trunk. Indeed, truncal acne represents an additional burden for patients beyond that caused by facial acne alone, with specific and distinct effects on activities of daily living, self-esteem and, social activities.^14^^,^^16^ As part of a 2020 consensus project, the Personalising Acne: Consensus of Experts (PACE) panel aimed to identify unmet needs in truncal acne and use an expert consensus, combined with the best available evidence, to make recommendations pertaining to gaps in truncal acne diagnosis, classification, and management.

## Methods

### Expert panel

The expert panel consisted of 13 dermatologists, from Canada (n = 2), France (n = 1), Germany (n = 1), Italy (n = 1), the United Kingdom (n = 1), and the United States (n = 7). Two chairpersons from the main panel oversaw the process and were involved in panel selection and Delphi design. The panelists were selected based on their expertise in acne and their reach in North America and Europe.

### Modified Delphi process

**Fig 1:**
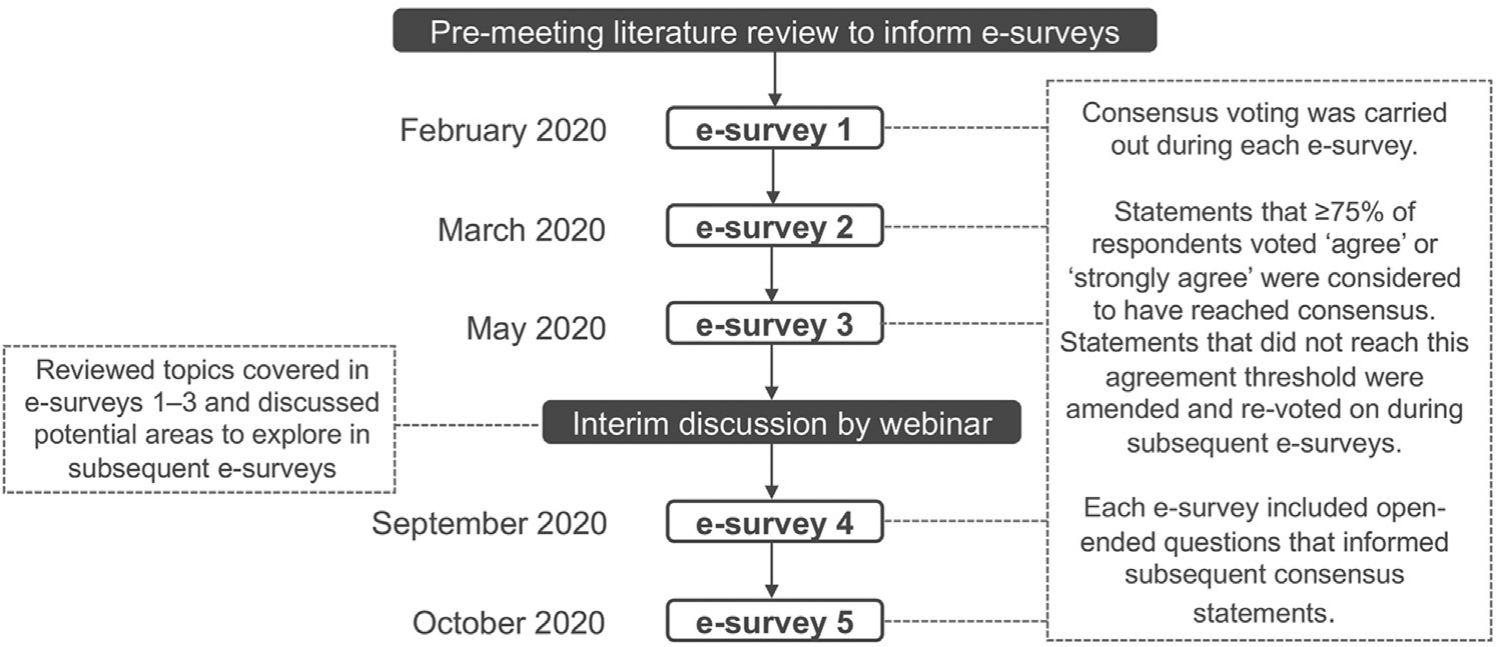
The Personalising Acne: Consensus of Experts modified Delphi process.

A modified Delphi process, consisting of a series of 5 e-surveys and an interim group webinar between the third and fourth e-survey, was used (Fig 1). An initial literature search was conducted to identify gaps in the clinical management of acne and the need to make recommendations that incorporate all presentations of acne. The search process, which is outlined in detail in Supplemental Material (available via Mendeley at https://data.mendeley.com/datasets/cnffzf3j4v/2), included an audit of acne clinical guidelines for Europe, the United States, and Canada^11^, ^12^, ^13^ to identify research gaps, followed by an additional literature assessment of the relevant literature to address key clinical management questions associated with the gaps identified in the audit. The searches were performed using the PubMed Central and Cochrane databases and limited to a period from January 2016 to present; the searches included only English-language articles. For the truncal acne management questions, the search terms included the word “acne” in combination with the following words: trunk, truncal, and treatment. Articles were excluded if they were on non-human animals, in vitro or ex vivo studies, or did not contribute to the research questions. A total of 36 articles specific to truncal acne were retrieved at the end of the search, and after applying predefined exclusions, 11 were used to address the research questions relating to truncal acne (Supplemental Material). The quality of evidence was rated using the grading of recommendations assessment, development and evaluation (GRADE) methodology^17^ and used to guide the e-survey content. Two independent raters (Mr Pickford and Dr Harris) performed a separate grade classification of the evidence, with discrepancies resolved by a third rater (Dr Hughes) with experience in the use of the grading of recommendations assessment, development and evaluation methodology. The final results were approved by the cochairs.

### E-survey development and administration

Consensus statements were structured to assess the level of agreement using the following response range: “strongly disagree,” “disagree,” “agree,” “strongly agree,” or “unable to answer.” A consensus was defined as ≥75% of the panelists voting “agree” or “strongly agree.” Some questions were posed as multiple-choice questions for which several responses could be selected; the results of these questions were presented as a consensus when chosen by ≥75% of the panelists. Some questions were open ended to allow for the development of consensus statements in a subsequent round of voting. A virtual interim meeting was held after the third e-survey to review topics covered to date and discuss potential areas to explore in the subsequent surveys. The e-surveys were programmed and administered, and the responses were collated by Ogilvy Health UK in order to maintain blinding. Truncal acne was 1 of 4 major topics explored in the e-surveys and virtual interim meeting and will be the focus of this current manuscript. Acne sequelae, longitudinal management, and patient types were also covered and will be reported in subsequent publications.

## Results

### Definition of consensus recommendations

The result of the consensus statement voting is given in parentheses (eg, 12/13 voted “agree” or “strongly agree”). All 13 experts completed all 5 surveys. Some panel members occasionally voted “unable to answer”; these votes were not included in the denominator. The full statements are available in Supplemental Material. The elements that were considered but not voted on are included in the “Discussion points” section below.

### Baseline demographics

All 13 dermatologists included in the PACE panel manage patients with truncal acne. For a majority of them (85% [n = 11]), ≤25% of their patients present with truncal acne alone. Just under half of the panelists (46% [n = 6]) indicated that 26%-50% of their patients present with combined facial and truncal acne. The other 7 (54%) panelists approximated that 51%-75% of their patients have combined facial and truncal acne.

When asked whether the panel considered clinical practice guidelines useful for the evaluation and management of acne in different anatomic locations, 69% (n = 9) and 62% (n = 8), respectively, did not find them useful, and 8% (n = 1) and 15% (n = 2), respectively, did find them useful.

### Identifying patients with truncal acne

The gaps and recommendations for identifying patients with truncal acne are provided in Table I.

**Table I:** Consensus gaps and a recommendation for identifying patients with truncal acne

Gaps • Not all patients report their truncal acne (13/13) • During clinical visits, patients often prioritize discussing their facial acne over discussing their truncal acne (12/13) • It is common for patients to only report their truncal acne if it is severe or particularly bothersome to them (11/13) • There is a need for guidance on factors to be consider when discussing truncal acne with patients (11/11)∗ Recommendation • Prompting patients to discuss their truncal acne during clinic visits can help identify those who might benefit most from treatment (13/13)

∗ Two participants selected “unable to answer.”

#### Discussion points

It was discussed that it might not be reluctance on a patient's part per se to initiate a conversation about their truncal acne but a desire to prioritize a discussion of their facial acne instead. However, there exists a cohort of patients who may be too embarrassed to report or proactively show acne on their trunk. Thus, it is important to proactively address this during consultations. The panelists reported that discussions about the management of truncal acne are typically initiated by themselves as the treating dermatologist (69% [n = 9]) rather than the patient or their guardian (23% [n = 3]; 8% [n = 1] selected “unable to answer”).

### Assessment and grading of truncal acne

The gaps and recommendations for the assessment and grading of truncal acne are provided in Table II.

**Table II:** Consensus gaps and recommendations for the assessment and grading of truncal acne

Gaps • There is a need for guidance on the assessment and grading of truncal acne (13/13) • There is a need for a standardized scale/tool to assess, grade, and monitor the severity of truncal acne (13/13) Recommendations • The severity of truncal acne should always be assessed independently of the severity of facial acne (13/13) • The essential clinical components to be included in a truncal acne grading tool or scale include body surface area involvement, size of lesions, degree or extent of inflammatory lesions, impact on patients quality of life, and extent of scarring (12/13) • A truncal acne grading scale or tool should be practical and easy to use in the clinic (13/13) • A new grading scale or tool to assess truncal acne should be developed based on input from patients to ensure that the elements that are important to them are captured (11/13)

#### Discussion points

It was highlighted that the severity of acne at different locations on the trunk (eg, chest and back) should be assessed independently of each other because they can be distinctly different. The panel reported using the following scales or tools for grading truncal acne in current clinical practice: investigator's global assessment, physician's global assessment (PGA), Leeds visual severity scale, Echelle de Cotation des Lésions d'Acné grading, comprehensive acne severity scale, and general mild, moderate, or severe categorization. It was discussed that most grading scales were developed for use in clinical trials and are not optimized for use in a clinical setting; in research, conversely, it can be difficult to combine both clinician- and patient-reported measures in 1 instrument that is also acceptable to regulatory authorities. A simplified version of these tools, which takes into account both the perspectives, may lend itself to use in practice.

### Impact of truncal acne on patients and treatment goals

The gaps and recommendations for the impact of truncal acne and treatment goals are provided in Table III.

**Table III:** Consensus gaps and recommendations for the impact of truncal acne and treatment goals

Gaps • Truncal acne can have a specific impact on patients, which is distinct from that of facial acne (13/13) • The common concerns or issues reported by patients specifically with regard to their truncal acne include the following: ○ Concerns about revealing skin in public (13/13) ○ Concerns about wearing clothes that reveal skin (12/13) ○ Difficulties with applying topical treatments to the back (11/13) Recommendations • Treatment goals should be personalized to the individual patient depending on the specific impact of acne in certain regions (13/13) • The specific burden of disease on an individual patient should influence the choice of treatment (13/13)

#### Discussion points

The panel considered truncal acne as a burden for all patients with truncal acne; however, it was noted that the burden could vary between patients. The visual analog scale (or similar) was suggested as a useful tool to assess factors related to the impact of the disease on patients and, thus, help inform treatment goals.

### Factors to be considered for the management of truncal acne

The gaps and recommendations for considerations for the management of truncal acne are provided in Table IV.

**Table IV:** Consensus gaps and recommendations for factors to be considered for the management of truncal acne

Gaps • There is a need for guidance on factors to be considered for the treatment and management of truncal acne (13/13) • There is a need for further evidence to support treatment efficacy in truncal acne (13/13) • There is a need for further evidence to support treatment safety in truncal acne (11/13) • There is a need to improve vehicles used for topical therapies for truncal acne (13/13) • There is a need to improve application methods for topical therapies for truncal acne (12/13) Recommendations • The treatment-related factors that should be considered when selecting a treatment for truncal acne include the following: ○ Type of acne, eg, nodules (13/13) ○ Efficacy of treatment (13/13) ○ Safety profile of treatment (10/13) ○ Practicality of applying topical treatment to the back (12/13) ○ Potential to bleach or stain clothing (10/13) • The patient-related factors that should be considered when selecting a treatment for truncal acne include the following: ○ Body surface area affected (13/13) ○ Previous treatment history (13/13) ○ Patient preference (12/13) ○ Location of truncal acne (ie, upper back, lower back, chest, and shoulders; 10/13) • The truncal acne types that require additional considerations for their management or treatment include the following: ○ Truncal acne at high risk of scarring (13/13) ○ Truncal acne with predominantly deep comedones (not related to hidradenitis suppurativa; 11/13) ○ Truncal acne associated with hidradenitis suppurativa (10/13) ○ Truncal acne with predominantly open comedones (10/13)

#### Discussion points

In the panel's opinion, the underlying pathophysiology of truncal acne is similar to that of facial acne. Any differences in response to treatment could be potentially attributed to the number and depth of sebaceous glands in either area, the relative thickness of the dermis, the role of exacerbating factors, and challenges related to the application of topical therapies (ie, physical application or when a patients runs out of a medication supply before a refill can be given). The examples of the treatments that panelists prescribe for truncal acne include topical benzoyl peroxide, topical and systemic retinoids, oral antibiotics (with appropriate cautions in terms of resistance), and oral isotretinoin. Addressing the inconvenience of application was highlighted as a key consideration while prescribing a topical treatment for truncal acne. In addition, acne associated with inflammatory bowel disease was also highlighted as requiring additional considerations for management and treatment.

The panel noted that patients' expectations of truncal acne treatment should be managed in a way similar to those of facial acne treatment, including counseling regarding expected timeframes and the importance of adherence to treatment. The panel highlighted the importance of patients having a realistic expectation of treatment efficacy and potential adverse events. It was acknowledged that patients might be willing to accept slower onset of efficacy on the trunk than on the face, although more data are needed to support this observation. Furthermore, although clearance is ideal, significant improvement may be an acceptable goal for many patients. It was noted that patients may be more willing to tolerate harsher or less cosmetically elegant products on the trunk than on the face.

The panel recommended that truncal acne be discussed with all patients with acne to identify those who might benefit most from treatment. In addition, it was noted that patients who already present with truncal scarring are at high risk of future scarring. Therefore, it is important to assess truncal acne independently of facial acne to determine its specific burden on individual patients and subsequently adjust their management plan, align treatment goals, and manage their expectations accordingly.

## Discussion

Truncal acne is a well-established condition that is common and burdensome for patients.^4^, ^5^, ^6^, ^7^, ^8^^,^^14^, ^15^, ^16^ In a recent cross-sectional online survey of 1309 patients with either combined truncal and facial acne or facial acne alone, the specific impact of acne was assessed in these locations using validated quality-of-life measures. Increasing severity of truncal acne was reported to increase adverse impact on the quality of life, irrespective of facial acne severity.^16^ This study highlights the need for improvements in patient care for truncal acne to lessen their burden. However, because of the lack of clinical evidence of truncal acne treatments and less mention in clinical practice guidelines,^8^ the need to provide physicians with practical advice on truncal acne management exists. Indeed, the paucity of information available to physicians about truncal acne may be reflected by the low proportion of panel members consulting them for guidance for the management of these patients (however, this may also be attributed to their clinical experience and knowledge). The PACE panel aimed at rectifying this situation by identifying key research gaps and providing recommendations for assessment and management beyond just treatment selection.

The panel confirmed that there is a gap in clinical research regarding the evidence of the efficacy and safety of truncal acne treatments. In the absence of sufficient high-quality evidence or clinical guidelines, the PACE panel identified various treatment- and patient-related factors to guide truncal acne management. The use of topical and oral antibiotics should be considered in light of the best evidence, including a means to minimize antibiotic resistance.^11^, ^12^, ^13^ The panel highlighted the need for better application methods and vehicles for topical treatments. Patient preference for vehicles for topical treatment is relevant to treatment adherence;^18^ with treatment adherence in patients with acne known to be an issue (especially for those prescribed topical treatments)^8^ and a major reason for treatment failure,^18^, ^19^, ^20^, ^21^, ^22^, ^23^, ^24^ this is an area that is critical to be explored further.

The PACE panel also identified the need for a standardized truncal acne grading scale or tool and identified several clinical components and features that are essential to be incorporated. The clinical guidelines do not advise on how to assess or grade truncal acne, and in the absence of a “gold standard” for the assessment of acne,^25^^,^^26^ physicians and clinical researchers may use simplified methods of “mild,” “moderate,” and “severe” grading^25^ or methods that focus on primary lesions (eg, comedones, papules, and nodules) or signs of a secondary change (eg, sequelae).^26^ Only a handful of the existing acne grading scales are inclusive of truncal acne: the Leeds system, global acne grading system, and comprehensive acne severity scale.^27^, ^28^, ^29^ However, none is exclusive to truncal acne.^8^ In addition, the Leeds system is not validated and only considers inflammatory lesions. Investigator's global assessment and PGA scores have been used in clinical studies of truncal acne in patients presenting with acne on the trunk and face,^30^, ^31^, ^32^ and in addition, the PGA was used to assess efficacy in 2 recent registration studies that resulted in Food and Drug Administration's approval of trifarotene,^10^ indicating the current Food and Drug Administration endorsement of the PGA as an appropriate clinician-reported outcome measure for truncal acne in clinical trials. The development of a standardized scale or tool for the assessment of truncal acne was beyond the scope of this consensus and would require a thorough development, evaluation, validation, and reliability-testing process. This represents an area for future evaluation.

This project incorporated the opinions of a global panel of dermatologists who achieved a consensus using a modified Delphi process. The limitation of the latter is that it primarily relies on expert opinion, necessary because of the absence of evidence.^33^, ^34^, ^35^ With current evidence of truncal acne limited across the spectrum of management considerations (assessment, grading, treatment efficacy, safety, etc), this method provides a systematic, egalitarian method to develop consensus recommendations to improve patient care.^36^ A particular strength of the Delphi process is that the group size does not depend on statistical power; instead, the group is selected for expertise,^37^^,^^38^ with the number of panelists within the recommended range of 10-18.^37^ The blinded voting reduced the potential for bias in the Delphi voting process.

An important limitation to be considered overall is that the identified gaps and recommendations proposed by the panel reflect physicians' perspectives on patient concerns regarding truncal acne. However, this could potentially differ from patients' perspectives. In addition, this perspective was of a group of expert dermatologists who are likely to see a more severe spectrum of patients with acne and/or those who are more burdened by it. The incorporation of the “patient voice” via direct patient involvement in the future updates of these recommendations or clinical practice guidelines would be of value to consider as an area of future work.

## Conclusions

The gaps that were identified by the PACE panel can guide further research on truncal acne. Furthermore, the recommendations made can provide a basis for local guideline development and help to improve the management of patients with truncal acne by facilitating increased attention during consultations.

## Conflicts of interest

All panel members received honoraria from Galderma for participating in this project. Dr Tan has acted as an advisor, consultant, investigator, and/or speaker and received grants/honoraria from Bausch, 10.13039/501100009754Galderma, Pfizer, Almirall, Boots/Walgreens, Botanix, Cipher Pharmaceuticals, 10.13039/501100009754Galderma, Novan, Novartis, Promius, Sun Pharma, and Vichy. Dr Alexis has received grant/research support from LEO Pharma, 10.13039/100004336Novartis, Almirall, Bristol-Myers Squibb, Amgen, Menlo, 10.13039/501100009754Galderma, Valeant (Bausch Health), Cara, and Arcutis; has acted as a consultant for LEO Pharma, Novartis, Menlo, Galderma, Pfizer, Sanofi-Regeneron, Dermavant, Unilever, Beiersdorf, Valeant, L'Oreal, Bristol-Myers-Squibb, Menlo, Scientis, Bausch health, UCB, Foamix, Cassiopea, Arcutis, Janssen, Allergan, Almirall, AbbVie, and Sol-Gel; and has acted as a speaker (unbranded) for Regeneron, SANOFI-Genzyme, Pfizer, and AstraZeneca. Dr Baldwin has acted as an investigator, consultant, and/or speaker for Almirall, Bausch, Cassiopea, EPI Health, Galderma, La Roche-Posay, L'Oreal, Mayne Pharma, Sol-Gel, Sun Pharma, and Vyne. Dr Beissert has acted as an advisory board member for AbbVie Deutschland GmbH & Co KG, Actelion Pharmaceuticals Deutschland GmbH, Amgen GmbH, Celgene GmbH, Galderma Laboratorium GmbH, Janssen-Cilag GmbH, LEO Pharma GmbH, Lilly Deutschland GmbH, Novartis Pharma GmbH, MSD Sharp & Dohme GmbH, Menlo Therapeutics, Sanofi-Aventis Deutschland GmbH, Pfizer Pharma GmbH, and UCB Pharma GmbH and has received speaker honorarium from Novartis Pharma GmbH, AbbVie Deutschland GmbH & Co KG, MSD Sharp & Dohme GmbH, Pfizer Pharma GmbH, Janssen-Cilag GmbH, Galderma Laboratorium GmbH, Celgene GmbH, La Roche-Posay Laboratoire Pharmaceutique, Actelion Pharmaceuticals Deutschland GmbH, GlaxoSmithKline GmbH & Co KG, Bristol-Myers Squibb GmbH & Co KGaA, Sanofi-Aventis Deutschland GmbH, Almirall-Hermal GmbH, and Sandoz/HEXAL AG. Dr Bettoli has acted as a consultant, advisory board member, and research investigator and received honoraria from AbbVie, Baiersdorf, Bioderma, Biogena, Difa-Cooper, Galderma, GSK, ICF, LEO Pharma, L'Oreal, Meda, Menarini – Relife, Mylan, Novartis, Pharcos-Biodue, UCB Pharma and received research support (funds to institution) from AbbVie. Dr Rosso has acted as a research investigator, consultant, and/or speaker for Almirall, Bausch Health (Ortho Dermatology), BiopharmX, EPI Health, Galderma, LEO Pharma, Mayne Pharma, Sol-Gel, Sonoma, Sun Pharma, and Vyne Therapeutics (Foamix). Dr Dréno has acted as a consultant for Galderma. Dr Stein Gold has acted as an investigator/advisor and/or speaker for Galderma, Ortho Derm, Sun Pharma, Sol-Gel, Foamix, Novartis, and Almirall. Dr Harper has acted as a consultant for Almirall, BioPharmX, Cassiopea, Cutera, EPI, Foamix, Galderma, Ortho, Sol-Gel, and Sun Pharma. Dr Lynde has acted as a principal investigator, speaker, and consultant for Cipher Pharma, Bausch Health, Galderma, Johnson & Johnson, GSK, and Valeant. Dr Thiboutot has acted as a consultant for Cassiopea, Galderma, and Novartis. Dr Weiss has acted as an investigator/advisor and/or speaker for Galderma, Ortho Derm, Foamix, Novartis, Almirall, Dr. Reddy's, and EPI Health. Dr Layton has acted as an advisor or consultant, been a chief investigator for research (funded to institution), and/or received honoraria for unrestricted educational events from Galderma, La Roche-Posay, L'Oreal, Cipher, Proctor and Gamble, Almirall, GSK, and Origimm.
